# Characterization of the HDAC/PI3K inhibitor CUDC-907 as a novel senolytic

**DOI:** 10.18632/aging.204616

**Published:** 2023-03-28

**Authors:** Fares Al-Mansour, Abdullah Alraddadi, Buwei He, Anes Saleh, Marta Poblocka, Wael Alzahrani, Shaun Cowley, Salvador Macip

**Affiliations:** 1 Mechanisms of Cancer and Aging Laboratory, University of Leicester, Leicester LE1 7RH, United Kingdom; 2 Department of Molecular and Cell Biology, University of Leicester, Leicester LE1 7RH, United Kingdom; 3 Clinical Laboratory Sciences Department, College of Applied Medical Sciences, Najran University, Najran 11001, Kingdom of Saudi Arabia; 4 The Ernest and Helen Scott Haematological Research Institute, University of Leicester, Leicester LE1 7RH, United Kingdom; 5 FoodLab, Faculty of Health Sciences, Universitat Oberta de Catalunya, Barcelona 08018, Spain

**Keywords:** senescence, senolytics, HDAC, PI3K, CUDC-907

## Abstract

The accumulation of senescent cells has an important role in the phenotypical changes observed in ageing and in many age-related pathologies. Thus, the strategies designed to prevent these effects, collectively known as senotherapies, have a strong clinical potential. Senolytics are a type of senotherapy aimed at specifically eliminating senescent cells from tissues. Several small molecule compounds with senolytic properties have already been identified, but their specificity and range of action are variable. Because of this, potential novel senolytics are being actively investigated. Given the involvement of HDACs and the PI3K pathway in senescence, we hypothesized that the dual inhibitor CUDC-907, a drug already in clinical trials for its antineoplastic effects, could have senolytic effects. Here, we show that CUDC-907 was indeed able to selectively induce apoptosis in cells driven to senesce by p53 expression, but not when senescence happened in the absence of p53. Consistent with this, CUDC-907 showed senolytic properties in different models of stress-induced senescence. Our results also indicate that the senolytic functions of CUDC-907 depend on the inhibitory effects of both HDACs and PI3K, which leads to an increase in p53 and a reduction in BH3 pro-survival proteins. Taken together, our results show that CUDC-907 has the potential to be a clinically relevant senolytic in pathological conditions in which stress-induced senescence is involved.

## INTRODUCTION

Ageing is a physiological condition defined in animals by a progressive functional impairment of most organs, driven at least in part by the accumulation of senescent cells [1]. Senescent cells, which have been shown to increase in aged tissues *in vivo* [2–4], are metabolically active but exhibit an irreversible and sustained cell cycle arrest [5–7]. Senescence can appear as a result of proliferation exhaustion (replicative senescence) or of different types of damage (stress-induced premature senescence, SIPS). The phenotype was first described when human diploid cells were observed to have limited proliferation ability *in vitro* [8], and since then it has been shown to be triggered in response to many stresses, such as therapy-induced senescence (TIS) [9–12], oncogene-induced senescence (OIS) [13], mitochondrial dysfunction-associated senescence (MiDAS) [14] or oxidative stress-induced senescence [15]. All these events cause a permanent cell cycle arrest via signalling pathways such as p16^INK4a^/RB and p53/p21^CIP1^, which inhibit the check point kinases (CDKs) and lead to a hypophosphorylated Retinoblastoma protein (RB) [16–18].

Senescent cells exhibit distinctive morphological changes and molecular markers, including enlarged flattened shape, large nucleoli, expression of CDK inhibitors and anti-apoptotic proteins, release of the senescence-associated secretory phenotype (SASP), increased reactive oxygen species (ROS), presence of senescence-associated β-galactosidase and α-lipofuscin, senescence-associated heterochromatin foci (SAHF) and epigenetic modifications [8, 16, 19–21]. Despite this, they are difficult to identify and target *in vivo*, since no universal marker of senescence has yet been found [22]. Different strategies have been developed to reduce the negative effects of senescent cell accumulation in tissues, collectively known as senotherapies: senoblockers to prevent the formation of senescent cells [23], senolytics to specifically eliminate them [24, 25] and senostatics/senomorphics to modulate the secretion of disruptive factors [26, 27]. Due to their huge therapeutic potential, great efforts are being made to identify novel senotherapies that can be tested in clinical trials [28, 29].

Since senescent cells are resistant to apoptosis due to the activation of Senescent Cell Anti-Apoptotic Pathways (SCAPs) [30], the first senolytics were developed to remove these pro-survival signals and thus allow apoptosis to proceed [31, 32]. SCAPs include signalling networks associated with PI3K/AKT and BCL-2/BCL-xL, among others [30, 32–34]. Many malignancies have been shown to have elevated PI3K/AKT activity [35–37]. Moreover, the loss of the tumour suppressor PTEN, the negative regulator of the PI3K/AKT pathway, causes senescence via the mTOR pathway [38], since mTOR1 and mTOR2 can inhibit MDM2 and activate p53 to promote cell cycle arrest [38].

Epigenetic alterations play an important role in the ageing process and the establishment of the senescent phenotype [20, 21, 39]. For instance, global hypomethylation of DNA is observed in replicative senescence *in vitro* [40] and in *in vivo* models [41–43]. Additionally, human diploid cells in culture showed an age-related decline in the rate of histone acetylation [44], and senescence caused by chemotherapy was associated with a reduction in histone-3 lysine-56 acetylation (H3K56ac) [45]. Hypoacetylation is also observed in senescent cells in different organs in humans [46, 47].

Histone acetylation is regulated by histone acetyltransferases (HATs) and histone deacetylases (HDACs) [15]. HDACs are a group of enzymes that deacetylate lysine residues in histone and non-histone proteins [48]. There are 18 distinct mammalian HDACs identified, which have been divided into four classes: Class I (HDAC1, 2, 3 and 8), subclass IIa (HDAC4, 5, 7 and 9) and subclass IIb (HDAC6 and 10), Class III (SIRT1–7), and Class IV (HDAC11) [49–51]. HDACs have been shown to play an important role in the establishment and maintenance of senescence [46, 47, 52–54]. For instance, HDAC4 has been involved in the induction of senescence [53] and may play a role in hypertension and cardiovascular diseases by modulating vascular senescence [52]. Additionally, HDAC6 could enhance SIPS in the retinal vasculature, which is involved in diabetic retinopathy [46]. Moreover, HDAC9 contributes to the development of adipose tissue senescence and can thus play a role in obesity-related metabolic diseases [47].

HDAC inhibitors (HDACi) are a series of small molecules that target the active site of different HDACs and inhibit their activity, which has shown a clinical effect on certain malignancies [55]. p53 activity can be modulated by acetylation [56, 57], among other post-translational alterations, and HDACi have been shown to facilitate p53 hyperacetylation, enhance p53 stability, decrease expression of anti-apoptotic genes and upregulate pro-apoptotic genes [58–61]. Interestingly, HDACi increase longevity [62–64] and ameliorate age-related pathologies [65, 66]. For example, exposure to the licensed HDAC inhibitor, sodium butyrate, can result in hyperacetylation of histone H4 at lysine 16 residue (H4K16), a reduction in the percentage of senescent cells and an extension of lifespan in Zmpste24^-/-^deficient mice, a model of accelerated ageing [64]. Moreover, HDAC inhibitor Panobinostat has been shown to have a senolytic effect [67].

In view of these findings, we hypothesized that CUDC-907, a heterobifunctional molecule that inhibits both HDAC (class I and II) and PI3K (class Iα, β, and δ) that has been shown to be well tolerated when given orally and to have antineoplastic activity [68], could have senolytic properties. We tested its effects on different cellular models of senescence and our results suggest that CUDC-907 could indeed be used as a senotherapy due to a selective specificity at very low concentrations for killing cells induced to senesce by p53. This could have therapeutic potential in diseases in which SIPS has a pathogenic role.

## RESULTS

### Selective sensitivity to CUDC-907 of cells undergoing p53-induced senescence

**Figure 1:**
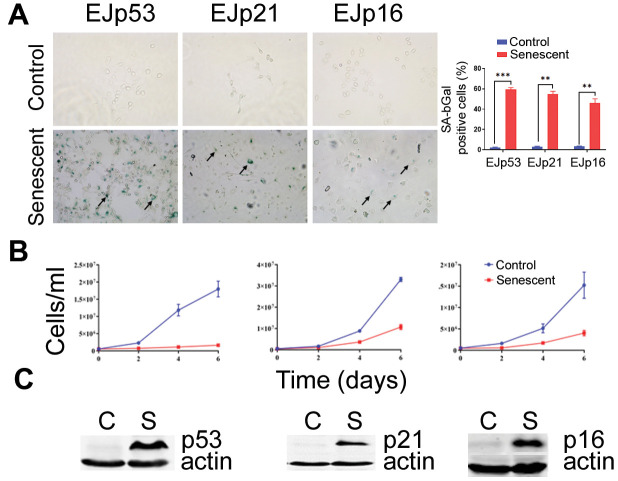
**Induction of senescence in the EJ models.** (**A**) Representative images of the SA-β-Gal staining of EJp53, EJp21 and EJp16 uninduced (Control, proliferating) or 6 days after tet removal to induce the expression of p53, p21 or p16, respectively (Senescent). Black arrows point to examples of senescent cells. The graph on the right is a quantitation of SA-β-Gal positive cells of three independent experiments. **, P < 0.01; ***, P < 0.001. (**B**) Cell counting of the same cell lines over a period of six days since tet removal. Three independent experiments were performed in duplicates and the averages and standard deviation (SD) were plotted in the graphs. (**C**) Representative Western blots of lysates of the same cell lines showing expression of the proteins induced by tet removal in each cell line. Actin was used as a loading control.

**Figure 2:**
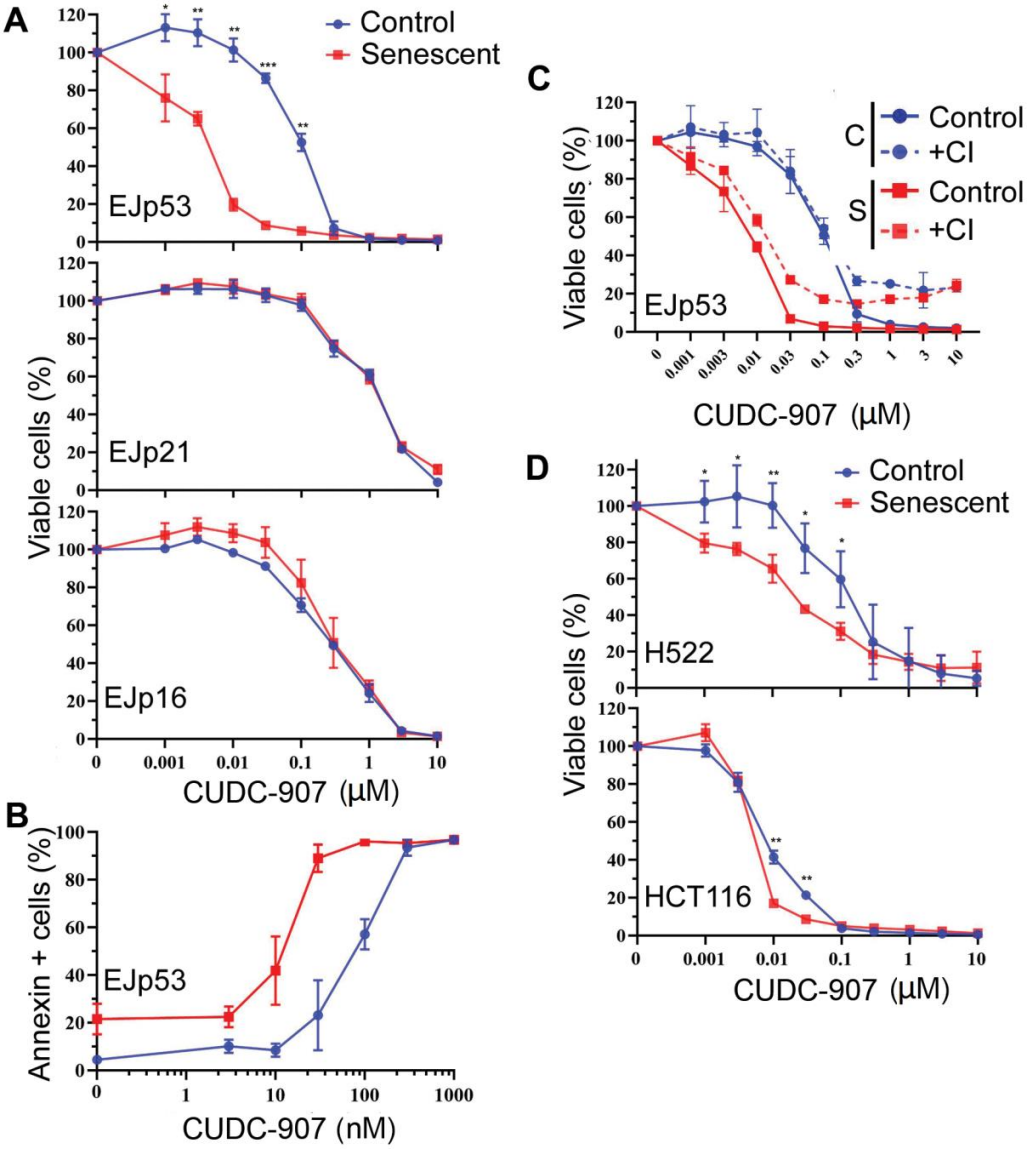
**CUDC-907 has senolytic effects in different models of cellular senescence.** (**A**) Cell viability of control (proliferating, blue) and senescent (6 days after tet removal, red) EJp53, EJp21 and EJp16 cells after treatment with different concentrations of CUDC-907 for 72h, as measured by a CTG assay. (**B**) Induction of apoptosis by different concentrations of CUDC-907 in control or senescent EJp53, as measured by Annexin V staining and FACs analysis. The percentages of Annexin V-positive cells are plotted. (**C**) Cell viability of control and senescent EJp53 after CUDC-907 treatment in the presence of DMSO (Control) or 10 μM of QVD-OPH (CI), as measured by a CTG assay. (**D**) Cell viability of control and senescent H522 and HCT116 72h after treatment with CUDC-907, as measured by a CTG assay. H522 were induced to senesce by exposure to 8 Gy of ionizing radiation and 6 days incubation. HCT116 were induced to senesce by exposure to 0.2 μM doxorubicin for 3 days. All values in this figure show mean ±SD of three independent experiments, and P values between each control and senescence pair are shown as *, P < 0.05; **, P < 0.01; ***, P < 0.001; ****, P < 0.0001.

In order to test the potential senolytic effects of CUDC-907, we took advantage of the genetic models of senescence, EJp53, EJp21 and EJp16, in which the p53 null bladder cancer cell line, EJ, is induced to senesce by a tetracycline(tet)-regulatable expression of p53, p21 or p16, respectively [69–71]. This system allowed to separately interrogate each of the main pathways involved in triggering senescence, i.e. p53/p21 and p16/Rb. Six days after tet was removed from the culture media, the majority of these cells were senescent, as confirmed by morphological changes and expression of senescence-associated-β galactosidase (Figure 1A), loss of proliferative capacity (Figure 1B) and expression of the respective markers (Figure 1C). Proliferating and senescent cells were treated with increasing concentrations of CUDC-907 for 72h. As shown in Figure 2A, CUDC-907 selectively decreased the viability of senescent EJp53 cells, but not EJp21 or EJp16, as measured by the rate of metabolic activity. This was highlighted by an IC_50_ of 3 nM in senescent EJp53, compared to 100 nM in proliferating controls (Supplementary Table 1). The effect was evident as early as 24 or 48 hours after treatment (Supplementary Figure 1). Interestingly, this was similar to the response observed when the same cells were exposed to BCL-2 inhibitor ABT-737, a known senolytic: senescent EJp53, but not EJp21 or EJp16, were sensitive to the drug (Supplementary Figure 2). These results indicate that cells in which senescence has been induced byp53 may be particularly sensitive to CUDC-907, suggesting for the first time a potential senolytic action of this drug similar to that of BCL-2 inhibitors, particularly in the context of SIPS, which is usually driven by a p53 response to stress.

### CUDC-907 induces apoptosis in senescent cells in the presence of p53

To further explore the senolytic effects of CUDC-907, we investigated how it reduced cell viability in senescent cells. As shown in Figure 2B, the Annexin-V staining, specific of cell death, suggested a significant increase in apoptosis of senescent EJp53 cells between 1 and 100 nM (with an IC_50_ of 10 nM, compared to 70 nM in proliferating controls). Of note, CUDC-907 induced death in 90% of senescent cells at 30 nM, while only 6% of the control proliferating cells died at this concentration, showing that it has an ample therapeutic window as a senolytic at low doses. Treatment with a pan-caspase inhibitor partially reduced the effects of CUDC-907 (Figure 2C), confirming that it induces death in senescent cells at least in part through the activation of the apoptotic cascade.

### CUDC-907 is senolytic in the context of stress-induced senescence

Our results show a strong senolytic effect of CUDC-907 in senescent cells that express p53, which suggests a possible effect in SIPS, a form of senescence in which p53 induction is central [72, 73]. To confirm this hypothesis in physiological models of stress-induced senescence, we used two cell lines subjected to levels of damage that can induce senescence: H522 (lung cancer with a mutated p53 [74]) treated with ionizing radiation and HCT116 (colon cancer with wt p53 [74]) treated with doxorubicin. Consistent with previous reports [24], we observed that a low dose of doxorubicin-induced senescence in HCT116 (Supplementary Figure 3). Similarly, H522 also entered a senescence-like state after being exposed to 8 Gy of ionizing radiation (Supplementary Figure 4). As shown in Figure 2D and Supplementary Table 1, CUDC-907 had a statistically significant specific effect in the senescent cells of these two models, although the window of action was smaller in HCT116. This confirms its role as a senolytic in SIPS, although it may not be dependent on the presence of functional p53.

### The senolytic effect of CUDC-907 depends on the inhibition of both HDAC and PI3K

**Figure 3:**
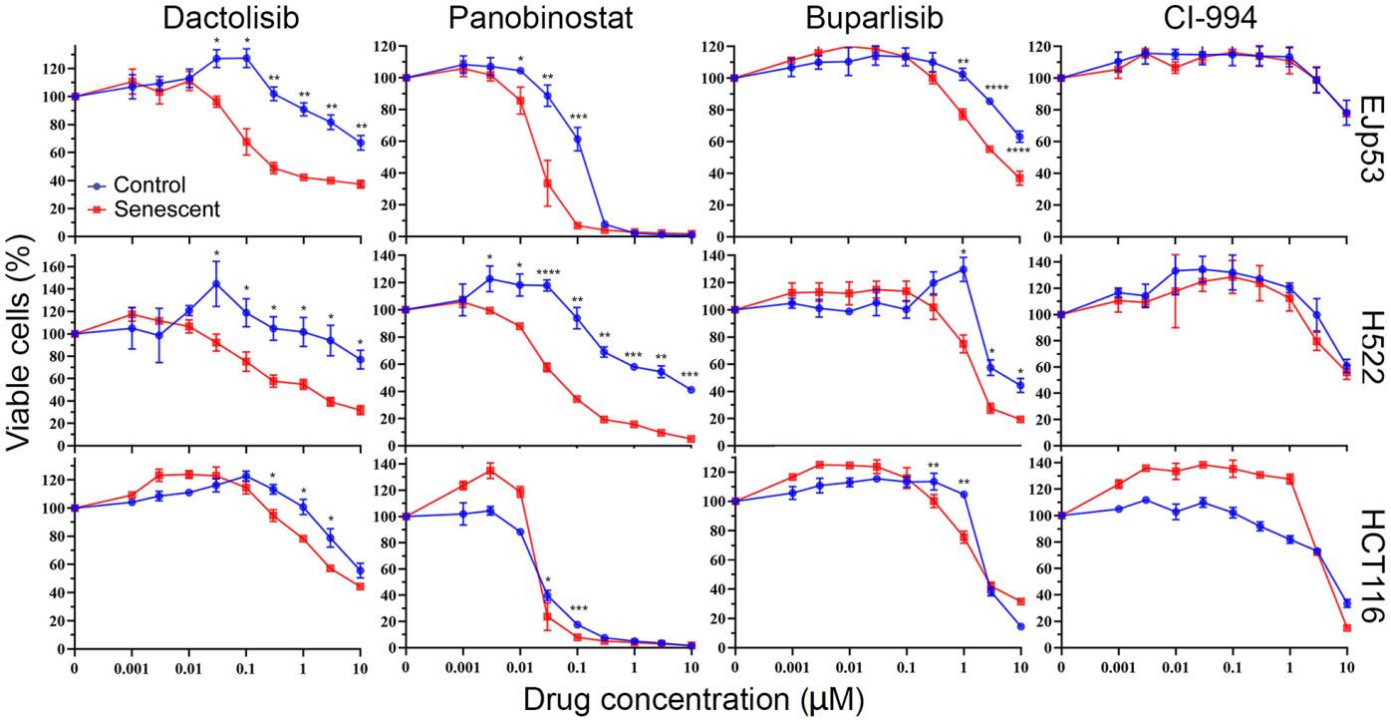
**Investigation of the pathways involved in the senolytic effects of CUDC-907.** Cell viability of control and senescent EJp53, H522 and HCT116 treated with different concentrations of dactolisib, panobinostat, buparlisib or CI-994 for 72h, as measured by a CTG assay. All values show mean ±SD of three independent experiments, and P values between each control and senescence pair are shown as *, P < 0.05; **, P < 0.01; ***, P < 0.001; ****, P < 0.0001.

CUDC-907 is a heterobifunctional molecule that contains chemical moieties that target both PI3K and HDAC [75]. To better understand the mechanisms by which it exerts its senolytic effect, we compared the response of senescent cells to specific drugs for these two pathways, with the goal of identifying which is most likely responsible for senolysis in CUDC-907. We found that, similar to CUDC-907, dactolisib (a PI3K inhibitor that can also inhibit mTOR) and panobinostat (a pan-HDAC inhibitor) had senolytic effects on EJp53 (Figure 3 and Supplementary Table 2) but not on EJp21 or EJp16 (Supplementary Figure 5). The effect was also significant, but at a lower magnitude, with buparlisib (a more specific PI3K inhibitor) but absent in CI-994 (which only inhibits class I HDACs). Dactolisib and panobinostat were also senolytic in H522, and had a minimal effect in HCT116 (Figure 3). Similar to EJp53, both senescent H522 and HCT116 showed a statistically significant but reduced sensitivity to buparlisib and no differences in CI-994 treatment, although the effect was less biologically relevant in HCT116. These data suggest that the inhibition by CUDC-907 of both PI3K and class II HDACs may contribute independently to its senolytic effects, although it is likely that other functions may also be playing a role and enhancing its ability to promote apoptosis in senescent cells.

### Potential mechanisms behind the senolytic effects of CUDC-907

**Figure 4:**
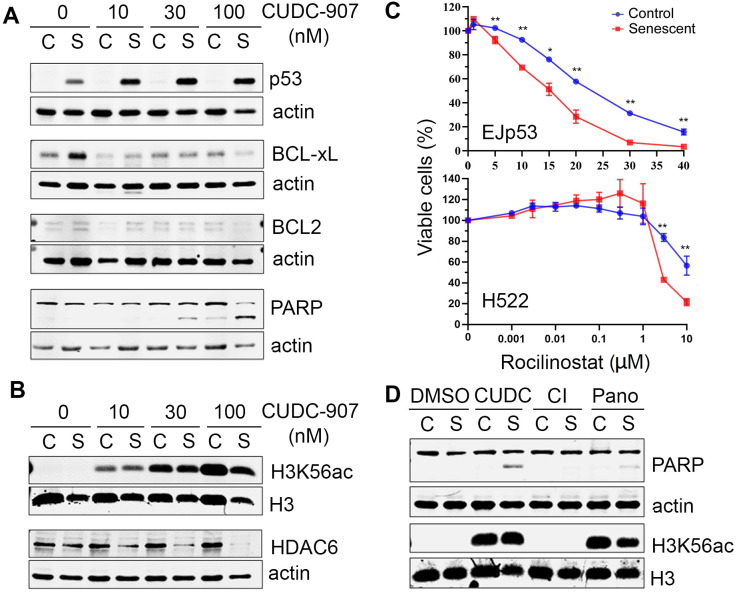
**Mechanisms of cell death induced by CUDC-907 in senescent cells.** (**A**, **B**) Representative Western blots of lysates of control (C) and senescent (S) EJp53 treated with different concentrations of CUDC-907 for 24h. Actin and H3 are used as a loading controls. (**C**) Cell viability of control and senescent EJp53 (top) and H522 (bottom) treated with different concentrations of rocilinostat for 72 hours. H522 were induced to senesce by exposure to 8 Gy of ionizing radiation and 6 days incubation. HCT116 were induced to senesce by exposure to 0.2 μM doxorubicin for 3 days. Values show mean ±SD of three independent experiments. (**D**) Representative Western blots of lysates of proliferating (C) and senescent (S) EJp53 treated with DMSO (control), 0.03 μM CUDC-907, 0.03 μM CI-994 (CI) or 0.03 μM panobinostat (Pano) for 24h. Actin and H3 are used as loading controls.

We next investigated in more detail the pathways involved in the senolytic effects of CUDC-907. As shown in Figure 4A, the expression of p53 was increased in EJp53 in the presence of CUDC-907, proportionally to the amount of apoptosis induced, as measured by PARP cleavage, which suggests p53 stabilization could be involved in triggering apoptosis by CUDC-907 in senescent cells. Moreover, pro-survival BH3 proteins BCL-xL and BCL2, which are upregulated in senescence [31, 32], were inhibited by CUDC-907, which may contribute to its senolytic effect. CUDC-907 also increased the acetylation of H3K56, as expected from an HDAC inhibitor, but selectively reduced the expression of HDAC6 in senescent cells (Figure 4B), suggesting that HDAC6 may have a pro-survival effect in these cells. Indeed, the HDAC6-specific inhibitor rocilinostat showed a particular toxicity for different types of senescent cells (Figure 4C and Supplementary Table 2), although not as striking as that of CUDC-907, which confirms that HDAC6 inhibition has a senolytic effect, in the presence or absence of functional p53, and may partially explain the mechanisms of action of CUDC-907. Of note, there were no changes in the expression of other class II HDACs, such as 7 and 9 (data not shown). We also found that the increase in H3K56ac levels was similar after CUDC-907 and panobinostat treatments, despite that the levels of apoptosis induced in senescent cells were not the same, as measured by PARP cleavage (Figure 4D), supporting the hypothesis that the HDAC inhibitory effect of CUDC-907 may not be sufficient to explain its senolytic activity. In summary, our data suggest that CUDC-907 has a strong senolytic effect that depends on its inhibition of PI3K and HDACs, particularly of HDAC6, and that this may be mediated by a concomitant increase in p53 activity, in cells in which is still wild type, and/or a reduction in pro-survival signals in senescent cells.

## DISCUSSION

In the past years, mounting evidence has proved that senescent cells accumulate in aged organisms and during many age-related diseases, including diabetes, Alzheimer’s and cancer [2–4]. More importantly, key studies have shown that preventing senescent cell accumulation minimizes their detrimental effects on tissue homeostasis, both in healthy and pathological conditions and improves lifespan and healthspan in animals [23, 76–78]. So far, senescent cells have been targeted in different experimental models using genetic methods [79] as well as senoblocker [23], senolytic [24, 25] and senostatic/senomorphic drugs [26, 27]. Some of these compounds are currently undergoing clinical trials [80], suggesting that the first application of a senotherapy in humans could be expected in the near future [81].

However, the forerunning senotherapies are mostly repurposed drugs with senolytic effects that are far from specific, and thus have the potential to trigger many off-target effects. This limits their intended therapeutic uses to diseases in which potentially serious side effects could be acceptable, like cancer or idiopathic lung fibrosis [82]. Because of this, great efforts are being invested in discovering novel senotherapies that could be more precise and effective than the first generation of drugs being tested at the moment [83].

It has been hypothesized that none of the known senolytics has a universal effect; instead, each of them is likely to only be effective in a subset of senescent cells. The sensitivity to these drugs would then be determined by the stimulus that induces senescence, the pathways that are preferentially activated, tissue of origin, presence of cofactors and modulators and other yet unknown factors. Thus, it would be important to expand the current catalogue of senolytics in order to define a wider library of compounds that could provide sufficient options for different key clinical uses.

In this context, we investigated CUDC-907, a compound that we suspected might have senolytic activity due to its chemical properties. Indeed, we found that cells that enter a stress-induced senescence (in response to chemicals or in the genetically-driven EJp53 model) were more sensitive to CUDC-907 than their proliferating counterparts, both when p53 was present and when it was mutated. Our results suggest that CUDC-907 can turn a p53-induced senescent response into apoptosis and may also induce cell death by other mechanisms in SIPS when p53 is not active, perhaps by removing pro-survival signals such as BCL-xL or BCL-2. However, when there is no direct stress response to prime the cells (i.e. in p21- and p16-induced senescence), this effect is lost. Interestingly, this is similar to how a well-studied senolytic of the BCL-2 inhibitor family works, suggesting that certain senolytics may have a common pathway of action that makes them more effective on particular types of senescence. This also provides novel insights on p53-mediated cell fate decisions [84], and could be particularly useful to eliminate cells that underwent SIPS, in which p53 is normally a driver [73], but it also could be a useful approach in cancer cells with mutated p53 induced to senescence after exposure to chemo or radiotherapy, which are believed to contribute to tumour relapse [11]. When we tested this hypothesis in different models of SIPS (two cancer cell lines of different origin and p53 status and two different damaging agents), we confirmed the senolytic effects of CUDC-907, but the results varied in range. This is consistent with the idea that senolytics will have increased specificity for certain types of senescent cells. It would be interesting to perform a wider screen of senescent models to determine in which ones CUDC-907 is more effective and thus design *in vivo* experiments that could inform of the potential clinical uses of the drug.

The mechanisms of induction of senescent cell death by CUDC-907 remain to be fully elucidated. Our data suggest that an apoptotic response may be triggered as a result of a combination of an increase in p53 activity and/or a decrease in pro-survival proteins. This is likely to happen in response to the simultaneous inhibition of different signalling pathways. Neither the inhibition of PI3K nor that of HDACs was sufficient to fully recapitulate the senolytic effects of CUDC-907, as specific inhibitors showed, suggesting that the combination of both had synergistic properties. It is possible that other functions of CUDC-907 may also contribute to this. Similarly, the mTOR-inhibiting activity of Dactolisib may be responsible for a more profound effect than Buparlisib, which lacks this effect despite also inhibiting PI3K. Interestingly, inhibition of HDAC6 could be important in the senolytic effect of CUDC-907, as HDAC6 levels were substantially decreased after treatment. Consistent with this, specific inhibition of HDAC6 activity had senolytic effects in our models, although not as striking as those of CUDC-907. HDAC6 has been shown to deacetylate p53 and inhibit p53-induced apoptosis, in part by preventing its acetylation at K120 [85–87], which could explain why its inhibition would increase p53 activity and thus push a senescent response towards apoptosis. Apart from HDAC6, it would be interesting to investigate which other HDACs may be protecting senescent cells against apoptosis, which could lead to the discovery of new senolytics. The fact that CI-994 alone was not effective, suggests that HDAC1/2/3 may not be involved.

Importantly, the senolytic effect of CUDC-907 was evident at nanomolar concentrations and had an ample therapeutic window, which is encouraging in terms of translating this discovery into a clinical tool. It should be taken into consideration that CUDC-907 is a drug that has already undergone clinical trials for other purposes and has been shown to be bioavailable and safe [68, 88, 89]. Nevertheless, it has off-target effects that could complicate its prescription as a senolytic, like all other known drugs in its class, as mentioned above. The development of second generation (or targeted) senolytics should provide a solution to this problem [90]. The main idea of this approach would be to couple an effective senolytic drug to a delivery system that would increase its specificity and thus avoid toxicity to non-senescent cells. For instance, we and others have shown that nanoparticles can carry a toxic cargo into senescent cells with great accuracy [25, 91]. Also, conjugating senolytics with galactose reduces their systemic toxicity by exploiting the high β-galactosidase content of senescent cells [92]. Recently, we have shown that antibodies directed against the senescent surfaceome [93] can be used as a basis of an antibody-drug conjugate that can deliver drugs into senescent cells [24]. All these strategies could help improve current senolytics and achieve their full therapeutic potential.

According to our results, CUDC-907 could be an interesting drug to be used as a senolytic, alone or as part of a targeted approach. For instance, it could be used as an adjuvant in oncological treatments to reduce the chances of relapse. Its strong effect at low concentrations and its wide therapeutic window are important assets that similar drugs do not possess, which puts CUDC-907 at the top of the list of novel senolytics to study. This is particularly relevant in view of the clinical tests already performed on this drug. However, further experiments would be needed to confirm the use of CUDC-907 as a senotherapy and find the best potential applications.

## MATERIALS AND METHODS

### Cell culture and senescence induction

EJ and HCT116 cells were grown in Dulbecco’s Modified Eagle’s Medium (DMEM), while H522 cells were cultured in Roswell Park Memorial Institute 1640 Medium (RPMI) (Thermo Fisher). Media were supplemented with penicillin-streptomycin (50 units/ml) and 10% Foetal Bovine Serum (FBS). EJp53 and EJp21 cells were cultured in complete culture media supplemented with 750 μg/ml geneticin and 100 μg/ml hygromycin, while EJp16 cells were maintained in media supplemented with 2 μg/ml puromycin and 100μg/ml hygromycin. To maintain EJ cells proliferating, 1 μM tetracycline was added to the culture media. To induce senescence, cells were trypsinized, washed with 1x phosphate buffered saline (PBS), and centrifuged at 244 g for three minutes. This step was repeated three times. Cells were cultured for a further 6 days in the absence of tetracycline to ensure the establishment of senescence. HCT116 cells were treated with 0.2 μM doxorubicin for 3 to 4 days to induce senescence. H522 cells were irradiated at 8Gy and cultured for 6 days to induce senescence. The drugs used were: CUDC-907 (APExBIO, #A4097), Dactolisib (Selleckchem, #BEZ235), Panobinostat (MedChemExpress, #404950-80), Buparlisib (APExBIO, #BKM120), CI-994 and Rocilinostat (Adooq Bioscience, #ACY-1215).

### Senescence-associated β-galactosidase (SA-β- gal) staining

Cells were stained according to previously described protocols [94]. Shortly, cells were washed in PBS before being fixed in 10% neutral buffered formalin for 5 minutes at room temperature, followed by another wash. After that, the cells were stained using staining buffer (1 mg/ml 5-bromo-4-chloro-3-indolyl-β-d-galactopyranoside (X-gal) in dimethylformamide, 150 mM Sodium Chloride, 2 mM Magnesium, 40 mM citric acid/sodium phosphate, pH 6.0, 5 mM Potassium Ferricyanide, 5 mM Potassium Ferrocyanide in distilled water). Plates were then incubated at 37° C in a non-CO_2_ incubator and photographed after 24 hrs.

### Western blotting

For protein extraction, collected cells were incubated in 200 μl of radioimmunoprecipitation assay (RIPA) lysis buffer (150 mM NaCl, 50 mM Tris HCl pH 8.0, 1% NP40, 0.1% SDS, 0.5% sodium deoxycholate), supplemented with 1 μg/ml phosphatase inhibitor cocktail 2 (Sigma-Aldrich) and protease inhibitor cocktail (Sigma-Aldrich), for 25 minutes on ice. They were then centrifuged at 14,000 g for 15 minutes at 4° C. A Bradford protein assay (Fermentas) was used to determine the protein concentrations in the supernatants. After adding 4X Laemmli buffer in a 1:4 ratio, samples were heated at 95° C for 7 min. Following that, 50 μg of total protein per sample were electrophoretically separated using SDS-polyacrylamide gels and then transferred to Immobilon-P membranes (Millipore). Membranes were then incubated with specific antibodies after blocking them in blocking buffer (5% BSA in PBS-Tween). Protein bands were detected and quantified with an Odyssey system (Li-COR, Lincoln, NE, USA). For histone extraction, the pellets after protein extraction were resuspended in 40% sulfuric acid standard solution (Hach) using the same volume that was used for protein extraction (200 μl) and incubated overnight at 4° C, followed by centrifuging and adding 4X Laemmli buffer. The antibodies used were: β-actin (Abcam, #ab8227), p53 (Santa Cruz Biotechnology, #sc-126), BCL2 (Dako, #M0887), Bcl-xS/L (Santa Cruz Biotechnology, #sc-271121), PARP (Cell Signaling Technology, #9542), H3K56Ac (Active Motif, #39281), H3 (Merck Millipore, #05-499), HDAC6 (Santa Cruz Biotechnology, #sc-28386).

### Cell viability and cell death analyses

6 x 10^4^ cells/ml were seeded into 96-well plates for 24 hours. The CellTiter-Glow (CTG, Promega) reagent was then applied to each well to evaluate the metabolic activity of the cells after adding the appropriate treatment for 72 hours, and luminescence was quantified using a Hidex Sense multimode microplate reader. Graphs were prepared using GraphPad Prism 9.0 software. Annexin V (Sigma-Aldrich) was used to calculate the percentage of apoptotic cells. Flow cytometry was used to determine the presence of apoptotic cells using a FACS Canto II cytometer (Becton Dickinson Biosciences) according to the manufacturer’s guidelines. The results were analysed using FACS Diva 6.1.3 software (BD Bioscience) and GraphPad Prism 9.0. For caspase inhibition, 6 x 10^4^ cells/ml were seeded into 96-well plates with QVD-OPH (Quinoline-Val-Asp-Difluorophenoxymethylketone, MedChemExpress), a pan-caspase inhibitor, at a final concentration of 10 μM. Cells were then treated with different concentrations of the appropriate drug for 72 hours, and the metabolic activity of the cells was measured as described above.

### Statistical analysis

GraphPad Prism 9.0 was used to perform two-tailed unpaired t-tests. Data from at least three independent experiments are represented in the figures as means and standard deviations. The statistical threshold for significance was selected at a *P*-value of 0.05: *, *P* < 0.05; **, *P* < 0.01; ***, *P* < 0.001; ****, *P* < 0.0001.

### Data availability statement

Data sharing is not applicable to this article as no datasets were generated or analysed during the current study.

## Supplementary Material

Supplementary Figures

Supplementary Tables
