# Supplementary material for: Characterization of the HDAC/PI3K inhibitor CUDC-907 as a novel senolytic
Source: Aging (Albany NY). 2023 Mar 28;15(7):2373–94. doi: 10.18632/aging.204616 (PMC10120895; doi:10.18632/aging.204616)
Supplement: Supplementary Figures [file aging-15-204616-s001.pdf]

SUPPLEMENTARY FIGURES

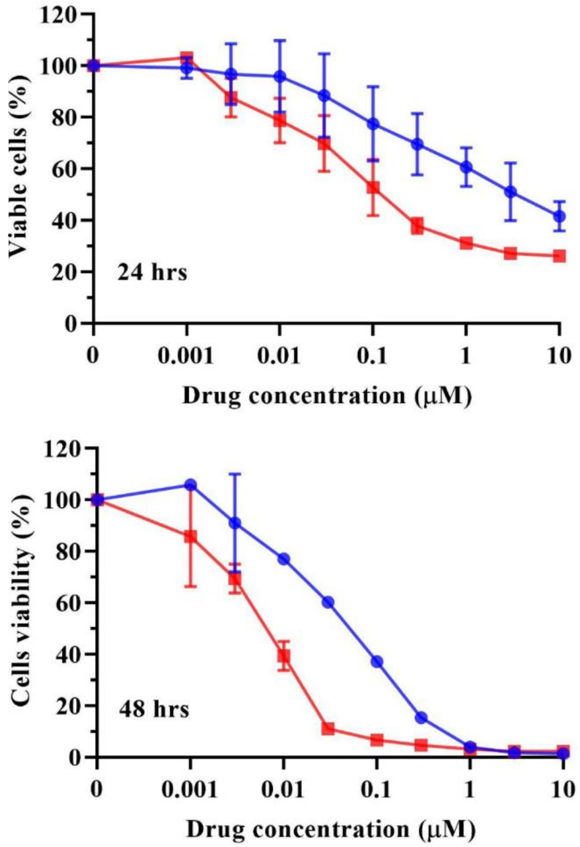

Supplementary Figure 1. CUDC-907 selectively reduces viability of senescent EJp53 at different time points. Cell viability of control (proliferating, blue) and senescent (6 days after tetracycline removal, red) EJp53 after treatment with different concentrations of CUDC-907 for 24h (top) or 48h (bottom). All values show mean ± SD of three independent experiments.

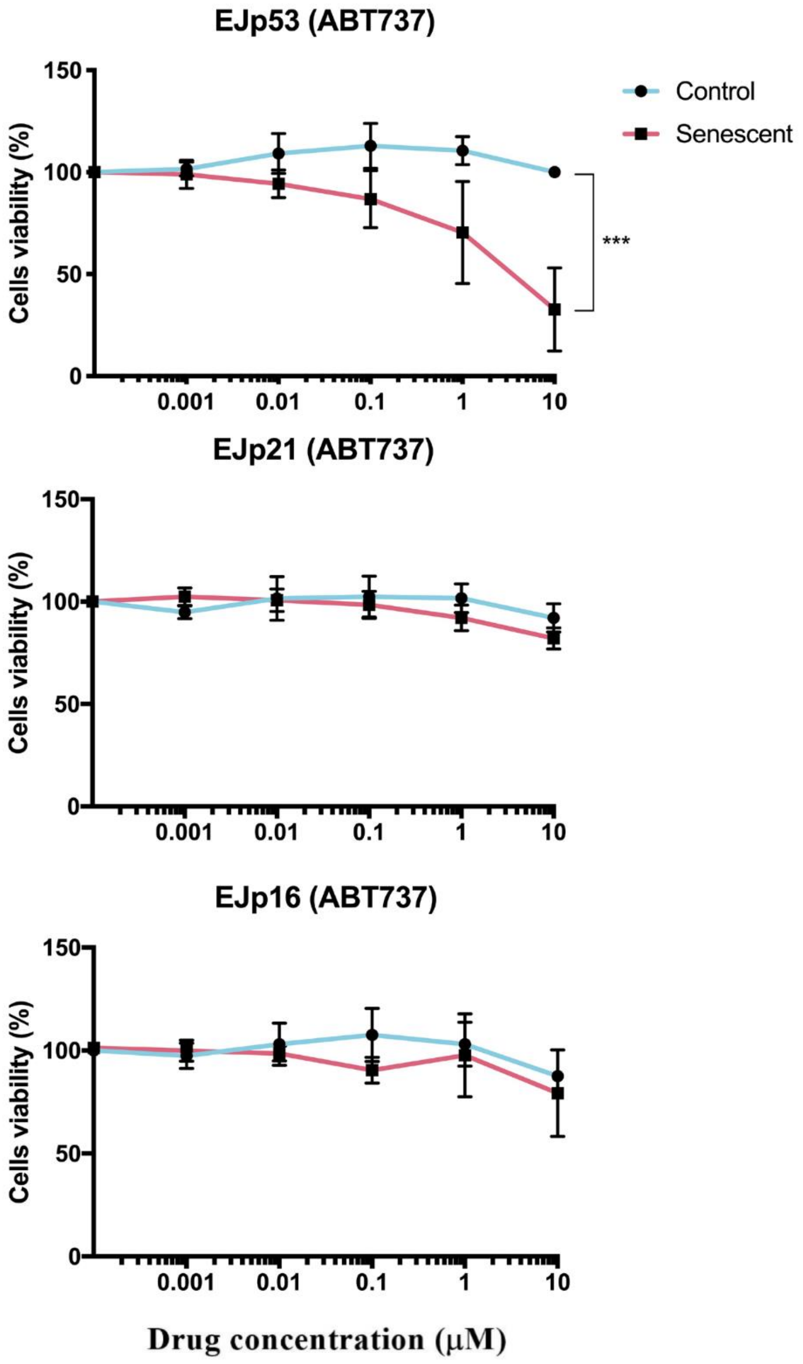

Supplementary Figure 2. Senolytic effects of ABT-737 on EJ cells. Cell viability of control (proliferating, blue) and senescent (6 days after tetracycline removal, red) EJp53, EJp21 and EJp16 after treatment with different concentrations of ABT-737 for 48h. All values show mean ± SD of three independent experiments. ***, $P < 0.001$.

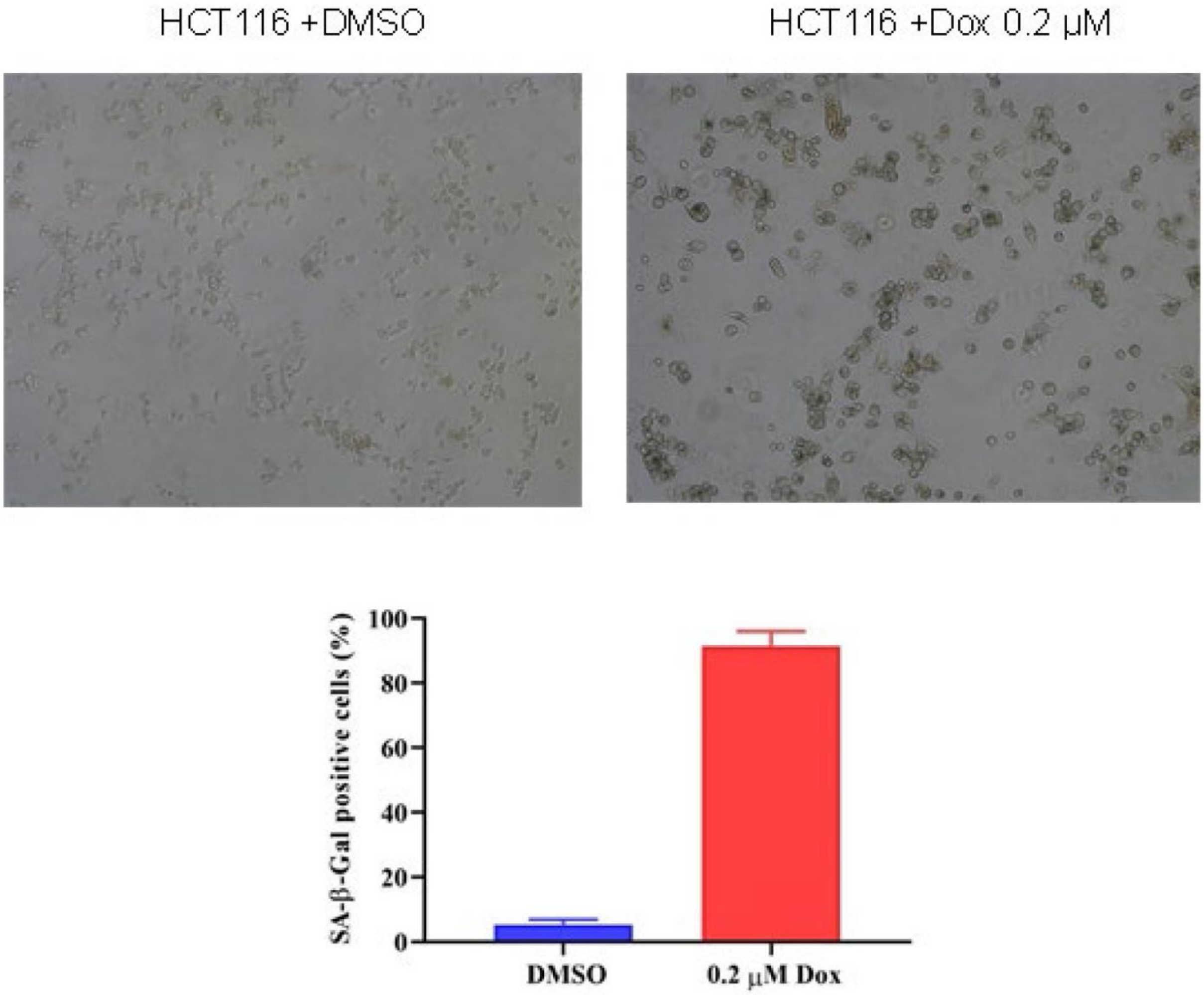

Supplementary Figure 3. Induction of senescence in HCT116. Top: Representative images of the SA-β-Gal staining of control (treated with DMSO for 3 days) or senescent (treated with 0.2 μM doxorubicin for 3 days). Bottom: quantitation of three independent experiments.

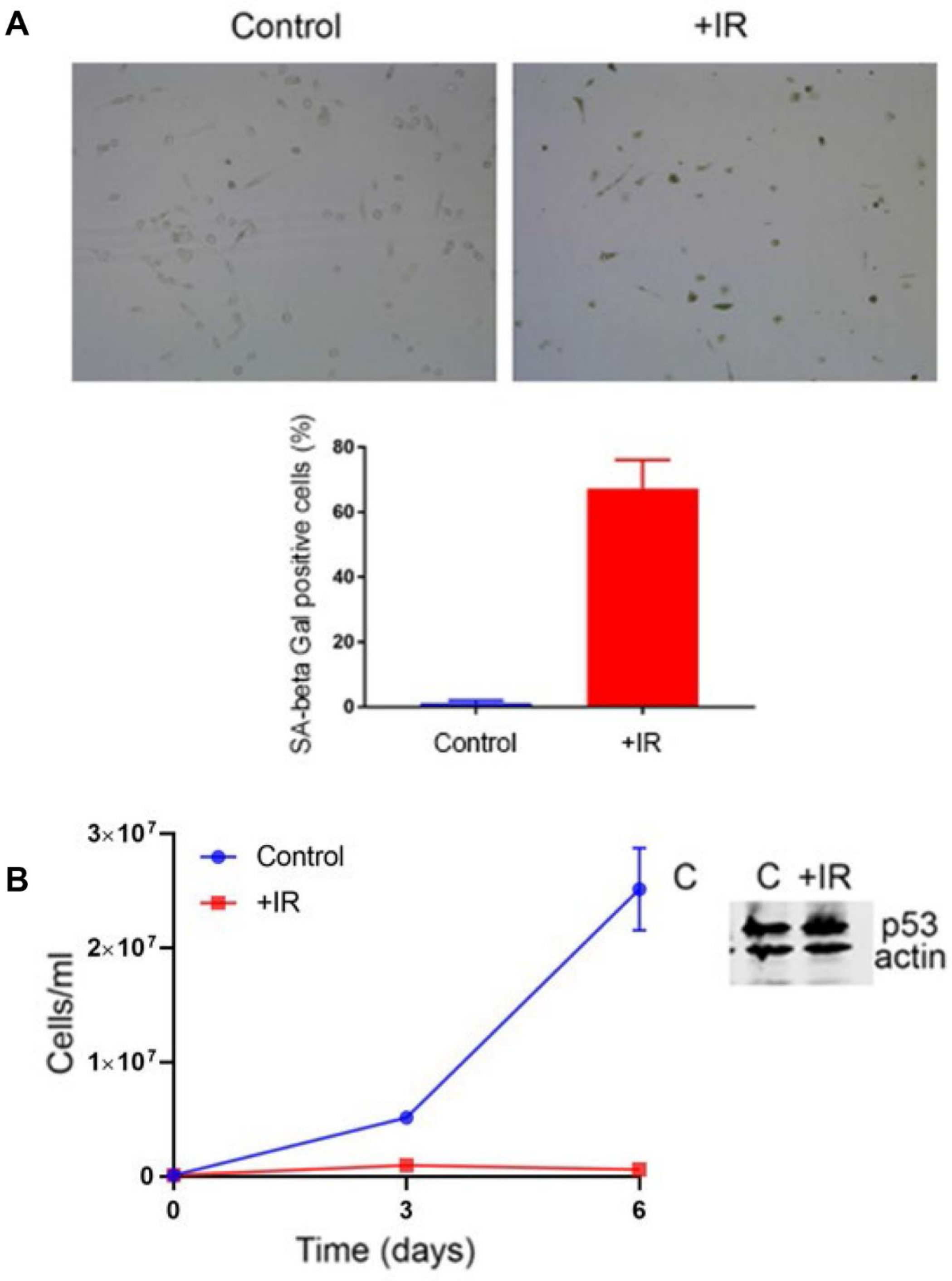

Supplementary Figure 4. Induction of senescence in H522. (**A**) Representative images of the SA-β-Gal staining of H522 cells treated (+IR) or not (Control) with 8Gy ionizing radiation for 6 days. (**B**) Cell counting of the same cell lines over a period of six days since treatment, showing the growth arrest of the irradiated cells. Three independent experiments were performed in duplicates and the averages and standard deviation (SD) were plotted in the graphs. (**C**) Representative Western blot showing induction of senescent marker p53 in lysates of the same cells. Actin is used as a loading control.

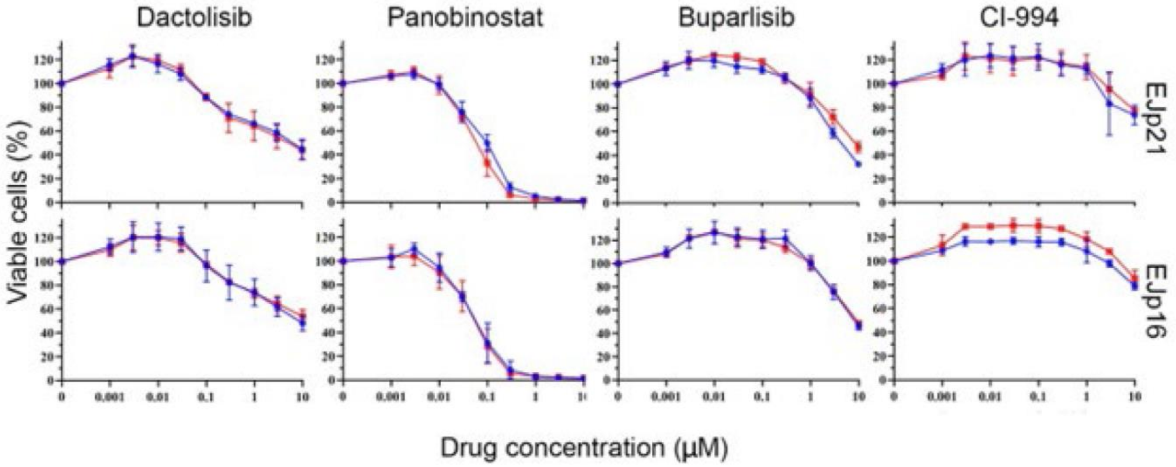

Supplementary Figure 5. Cell viability in EJp21 and EJp16. Cell viability of control and senescent EJp21 and EJp16 treated with different concentrations of dactolisib, panobinostat, buparlisib or CI-994 for 72h, as measured by a CTG assay. All values show mean ± SD of three independent experiments.
