# Supplementary material for: Characterization of the HDAC/PI3K inhibitor CUDC-907 as a novel senolytic
Source: Aging (Albany NY). 2023 Mar 28;15(7):2373–94. doi: 10.18632/aging.204616 (PMC10120895; doi:10.18632/aging.204616)
Supplement: Supplementary Tables [file aging-15-204616-s002.pdf]

SUPPLEMENTARY TABLES

Supplementary Table 1. IC_{50} values (drug concentration in which the metabolic activity was reduced 50%, in µM) of EJp53, EJp16, EJp21, H522 and HCT116 treated with CUDC-907, taken from the results shown in Figures 2A, 2C.

Cell line	CUDC-907 IC_{50} (µM)	
	Control	Senescent
EJp53	0.09984	0.003880
EJp21	1.161	1.218
EJp16	0.2781	0.3555
H522	0.1320	0.02417
HCT116	0.008709	0.005477

Supplementary Table 2. IC_{50} values (in μM) of EJp53, H522 and HCT116 treated with CUDC, dactolisib, panobinostat, buparlisib, CI-994 or roctilinostat, taken from the results shown in Figures 3A, 4C.

Cell line	Dactolisib IC_{50} (μM)	
	Control	Senescent
EJp53	18.10	0.9500
H522	21.18	1.320
HCT116	11.15	5.686

Cell line	Panobinostat IC_{50} (μM)	
	Control	Senescent
EJp53	0.1176	0.02236
H522	3.359	0.05717
HCT116	0.02726	~ 0.02741

Cell line	Buparlisib IC_{50} (μM)	
	Control	Senescent
EJp53	14.26	4.556
H522	7.014	1.938
HCT116	~2.878	3.083

Cell line	CI-994 IC_{50} (μM)	
	Control	Senescent
EJp53	14.72	14.43
H522	10.86	11.05
HCT116	5.881	4.648

Cell line	Roctilinostat IC_{50} (μM)	
	Control	Senescent
EJp53	22.25	14.24
H522	15.16	4.892
